# Effects of Cobalt Chloride, a Hypoxia-Mimetic Agent, on Autophagy and Atrophy in Skeletal C2C12 Myotubes

**DOI:** 10.1155/2017/7097580

**Published:** 2017-06-19

**Authors:** Rui Chen, Ting Jiang, Yanling She, Jiehua Xu, Cheng Li, Shanyao Zhou, Huijuan Shen, Huacai Shi, Shuang Liu

**Affiliations:** ^1^Guangdong Traditional Medical and Sports Injury Rehabilitation Research Institute, Guangdong No. 2 Provincial People's Hospital, 466 Xin Gang Zhong Road, Guangzhou 510317, China; ^2^Department of Radiology, The Third Affiliated Hospital, Sun Yat-sen University, 600 Tian He Road, Guangzhou 510630, China; ^3^Department of Nuclear Medicine, The Third Affiliated Hospital, Sun Yat-sen University, 600 Tian He Road, Guangzhou 510630, China; ^4^Department of Hematology, Guangdong No. 2 Provincial People's Hospital, 466 Xin Gang Zhong Road, Guangzhou 510317, China

## Abstract

**Background:**

Hypoxia-induced autophagy and muscle wasting occur in several environmental and pathological conditions. However, the molecular mechanisms underlying the effects of the hypoxia-mimetic agent CoCl_2_ on autophagy and muscle atrophy are still unclear.

**Methods:**

C2C12 myotubes were exposed to increasing concentrations of CoCl_2_ for 24 hours. Quantitative RT-PCR, Western blotting, and transmission electron microscopy were performed to confirm autophagy occurs. Autophagy proteins were measured to understand the molecule mechanisms. We also inhibited hypoxic autophagy and examined the changes in myogenin expression, myotubes formation, and apoptosis.

**Results:**

Our results showed that CoCl_2_-mimicked hypoxia upregulated the expression of the autophagy-related proteins LC3, HIF-1*α*, BNIP3, p-AMPK*α*, and beclin-1, whereas p62 and p-mTOR were downregulated. In addition, the autophagosome could be observed after CoCl_2_ induction. The expression of the autophagy-related E3 ligase parkin and the muscle-specific ubiquitin ligase atrogin-1 was increased by CoCl_2_. Inhibition of autophagy by 3MA increased myogenin expression and promoted myotubes formation and the percentage of cell death was decreased.

**Conclusions:**

Our results confirmed that CoCl_2_-mimicked hypoxia induced autophagy via the HIF-1*α*/BNIP3/beclin-1 and AMPK/mTOR pathways. Our results also revealed an important link between autophagy and muscle atrophy under hypoxia, which may help to develop new therapeutic strategies for muscle diseases.

## 1. Introduction

Oxygen plays a central role in cellular respiration and energy metabolism. However, hypoxia is common in the tissues of most individuals. Hypoxia-induced muscle wasting is a phenomenon frequently reported in several environmental and pathological conditions, such as exposure to high altitudes, prolonged immobilization, chronic obstructive pulmonary disease, exercise, and anemia [1–4]. However, the mechanism underlying the effects of hypoxia in skeletal muscle is still unknown.

Autophagy is a catabolic process that eliminates or recycles obsolete proteins and organelles via lysosomes to maintain cellular homeostasis. Autophagy occurs constitutively in skeletal muscle under many physiological conditions and becomes an important regulator in hypoxic environments, helping to maintain a balance between synthesis and degradation.

Hypoxia inducible factor-1 alpha (HIF-1*α*) is a transcription factor that controls hypoxia-induced autophagy by upregulating expression of its downstream proteins, such as Bcl-2 adenovirus E1B 19-kDa interacting protein 3 (BNIP3) [5]. BNIP3 then forms a stable homodimer complex that is inserted into the mitochondrial membrane, causing mitochondrial damage and triggering mitochondrion-dependent apoptosis [6]. Earlier reports suggested that BNIP3 plays a pivotal role in the loss of skeletal muscle mass and provides a potential therapeutic target in muscle wasting disorders and other diseases that involve autophagy [7]. Beclin-1 is a downstream target of BNIP3. BNIP3 hinders interaction between Bcl-2 and beclin-1, resulting in increased availability of beclin-1 and subsequent induction of autophagy [8].

Autophagy induction requires the activation of several signaling kinases. One potent autophagy activator is AMP-activated protein kinase (AMPK), a conserved serine/threonine kinase required for cellular growth and energy homeostasis [9]. Conversely, mammalian target of rapamycin (mTOR) is negatively regulating autophagy in mammalian cells [5]. When nutrients are scarce, AMPK phosphorylates and interacts with unc-51 like autophagy activating kinase 1 (ULK1). mTOR dissociates from ULK1 complex, freeing ULK1 to trigger autophagosome nucleation and elongation [10]. When nutrients are abundant, the mTOR complex 1 (mTORC1) binds to and phosphorylates ULK1 Ser757 and then disrupts the interaction between ULK1 and AMPK [11]. Although both AMPK and mTOR play important role in autophagy, their contribution to the induction of the autophagy in skeletal muscle under hypoxia has been only barely investigated so far.

The present study was performed to investigate the effects of cobalt chloride (CoCl_2_) as a mimic of hypoxia on autophagy and atrophy. We used CoCl_2_ to mimic hypoxia in vitro in the C2C12 mouse myoblast cell line. With regard to the molecular mechanisms involved, we examined the expression of HIF-1*α*/BNIP3/beclin-1 and AMPK/mTOR, which affected autophagy. To explore the effects of hypoxia on muscle mass degeneration further, we examined the expression of parkin and atrogin-1. Based on previous studies reporting both beneficial and detrimental effects of autophagy on muscle homeostasis, we inhibited autophagy under hypoxia in the C2C12 mouse myoblast cell line to examine the association between autophagy and atrophy, which may help in the development of new therapeutic strategies for muscle diseases.

## 2. Materials and Methods

### 2.1. Cell Culture

The mouse myoblast cell line C2C12 (Stem Cell Bank, Chinese Academy of Sciences) was cultured in DMEM high glucose (Gibco-BRL, Grand Island, NY) supplemented with 10% fetal bovine serum (Hyclone, Logan, UT), 100 U/mL penicillin, and 100 *μ*g/mL streptomycin in 5% CO_2_ at 37°C. Myoblasts were induced to form myotubes by incubation in DMEM containing 2% horse serum (Hyclone, Logan, UT) for another 5 days. Then, C2C12 myotubes were treated with CoCl_2_ at different final dilutions (10, 50, 100, or 200 *μ*M) for 24 hours. 3-methyladenine (3MA) (Selleck, Houston, TX) was cocultured with/without CoCl_2_ at a concentration of 5 mM for 24 h. Chloroquine (CQ) (Selleck, Houston, TX) was added to differential medium at a working concentration of 25 *μ*M for 6 h before collection of cells.

### 2.2. Real-Time Quantitative PCR

Total RNA was extracted from cells using TRIZOL reagent (Life Technologies, NY, USA), in accordance with the manufacturer's protocol. Aliquots of 1 *μ*g of RNA were reverse-transcribed to cDNA with PrimeScript™ RT Master Mix (Takara Biotechnology Co., Ltd., Otsu, Japan). SYBR® Green Mix (Takara Biotechnology Co., Ltd.) was used to determine the abundance of mRNA, and the results were expressed relative to 18S RNA. The primer sequences used for PCR were as follows:

18S forward: 5′-GTAACCCGTTGAACCCCATT-3′, reverse: 5′-CCATCCAATCGGTAGTAGCG-3′; HIF-1*α* forward: 5′-ACCTTCATCGGAAACTCCAAAG-3′, reverse: 5′-CTGTTAGGCTGGGAAAAGTTAGG-3′; BNIP3 forward: 5′-TGAATCTGGACGAAGTAGCTCC-3′, reverse: 5′-CAGACGCCTTCCAATGTAGATC-3′; LC3B forward: 5′-CGATACAAGGGGGAGAAGCA-3′, reverse: 5′-ACTTCGGAGATGGGAGTGGA-3′; myogenin forward: 5′-GGCAATGCACTGGAGTTCG-3′, reverse: 5′-AGCCGCGAGCAAATGATC-3′; atrogin-1 forward: 5′-GAGTGGCATCGCCCAAAAGA-3′, reverse: 5′-TCTGGAGAAGTTCCCGTATAAGT-3′; p62 forward: 5′-CACAGGCACAGAAGACAA-3′, reverse: 5′-CCGACTCCAAGGCTATCT-3′; parkin forward: 5′-ACCATCAAGAAGACCACCAAG-3′, reverse: 5′-GTTCCACTCACAGCCACAG-3′.

### 2.3. Western Blotting Analysis

C2C12 myotubes were lysed in RIPA buffer containing protease inhibitor and PMSF to extract the total protein. Equal quantities of proteins (20 *μ*g) were separated by 10%–12% SDS-PAGE and transferred onto PVDF membranes. The membranes were blocked with 5% nonfat milk and incubated with primary antibodies targeting HIF-1*α* (1 : 1000; Abcam, Cambridge, UK), BNIP3 (1 : 1500; Abcam), atrogin-1 (1 : 1000; Abcam), LC3B (1 : 1000; ABclonal, Woburn, MA), beclin-1 (1 : 2000; ABclonal), myogenin (1 : 500; Millipore, Billerica, MA), parkin (1 : 1000; CST, Danvers, MA), p62 (1 : 500; CST), p-mTOR (1 : 1000; CST), mTOR (1 : 1000; CST), p-AMPK*α* (1 : 1000; CST), and AMPK*α* (1 : 1000; CST) overnight at 4°C. The membranes were incubated with goat anti-mouse or anti-rabbit secondary antibody for 1 hour at room temperature. Band intensity was determined using a chemiluminescent imaging system (Tanon, Shanghai, China). Tubulin was used as a control for protein level quantification.

### 2.4. Transmission Electron Microscopy

Cell specimens were fixed in 2.5% glutaraldehyde and then postfixed in 1% osmium tetroxide, dehydrated through a graded ethanol series, and embedded in epoxy resin. Serial ultrathin sections were cut on an LKB-III ultratome (Leica, Wetzlar, Germany). Ultrathin sections were stained with uranyl acetate (Ted Pella, Redding, CA) and lead citrate (Ted Pella) and examined using an electron microscope (H7600; Hitachi, Tokyo, Japan) at an acceleration voltage of 100 kV.

### 2.5. Giemsa Staining

Cells were fixed in pure methanol for 10 minutes and then immersed in a freshly prepared working Giemsa stain solution (KeyGEN Biotech, Jiangsu, China) for 20 minutes, flushed with tap water, and left to dry last.

### 2.6. Detection of Necrosis and Apoptosis

An Annexin V-fluorescein isothiocyanate (FITC) apoptosis detection kit (Sony Biotechnology Co., CA, USA) was used to detect apoptosis in accordance with the manufacturer's instructions. The C2C12 myotubes were incubated with CoCl_2_ or 3MA for 24 hours. The cells were then digested with trypsin and washed twice with cold PBS. The cells were resuspended in 500 *μ*L of binding buffer. Then, 5 *μ*L of Annexin V and 5 *μ*L of 7-ADD were added to the cells and incubated in the dark for 15 minutes.

### 2.7. Statistical Analysis

Data are reported as the means±SEM. Statistical significance was assessed by one-way ANOVA between groups. When significant variations were found, Tukey's multiple comparisons test was performed. In all analyses, *P* < 0.05 was taken to indicate statistical significance.

## 3. Results

### 3.1. Cobalt Chloride Induced Autophagy in C2C12 Cells

**Figure 1:**
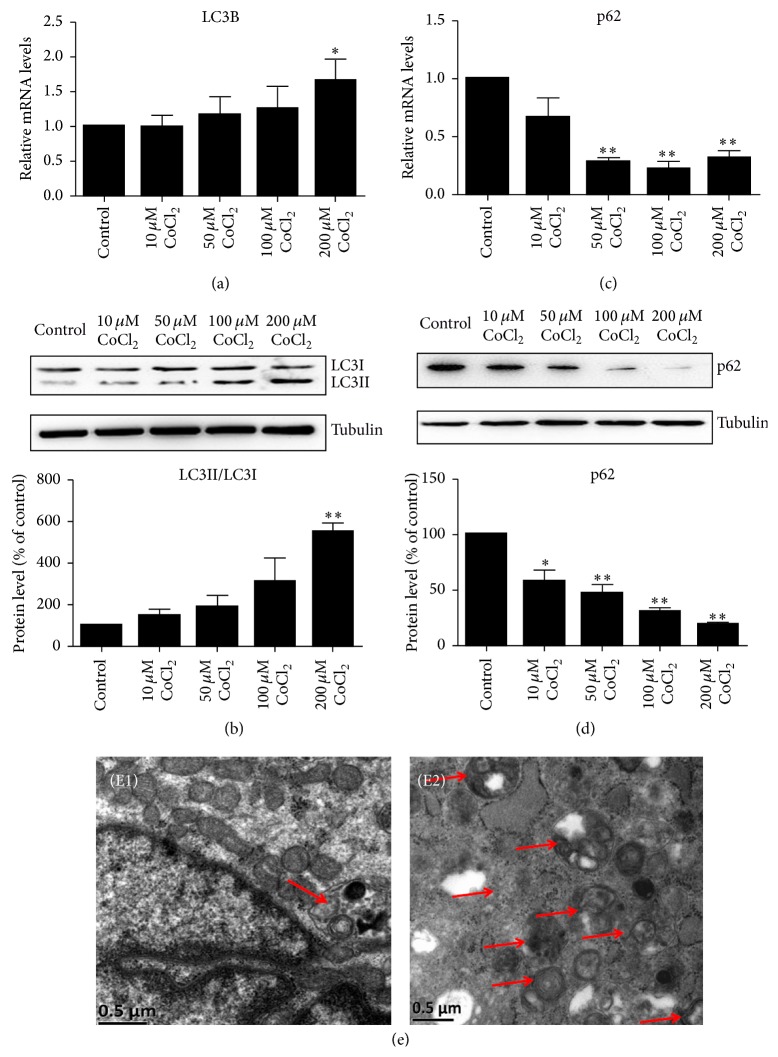
CoCl_2_ induced C2C12 myotubes autophagy. The C2C12 myotubes were incubated with different dilutions of CoCl_2_ (10, 50, 100, or 200 *μ*M) for 24 hours. QRT-PCR and Western blotting analysis were, respectively, used to determine the mRNA and protein levels of LC3 and p62 in C2C12 cells treated with CoCl_2_ (a–d). The bands were quantified using Image J and tubulin was used as the internal control. ^*∗*^*P* < 0.05 and ^*∗∗*^*P* < 0.01 compared to control group. (e) Transmission electron microscopy images of C2C12 cells showing increased numbers of autophagosomes in the CoCl_2_ (200 *μ*M) group (E2) compared to the normal group (E1). Scale bar: 500 nm. The red arrows mean autophagosomes.

To examine the effects of CoCl_2_ mimicking hypoxia on autophagy in C2C12 cells, we performed qRT-PCR and Western blotting analysis to determine the expression of LC3B and p62 at different concentrations for 24 hours. The results showed that CoCl_2_ dose-dependently increased LC3B mRNA (Figure 1(a)) and the ratio of LC3II/LC3I (Figure 1(b)). p62 is an autophagic adaptor protein which can be degraded during increased autophagy. In support of increasing LC3B-II protein, p62 was dramatically reduced in CoCl_2_ treatment groups (Figures 1(c) and 1(d)), which could be interpreted as an increase in autophagy flux. In addition, the autophagosome could be observed with CoCl_2_ treatment (Figure 1(e)).

### 3.2. The Real Effect of Cobalt Chloride Induced Autophagy Was Further Verified by 3MA and CQ

**Figure 2:**
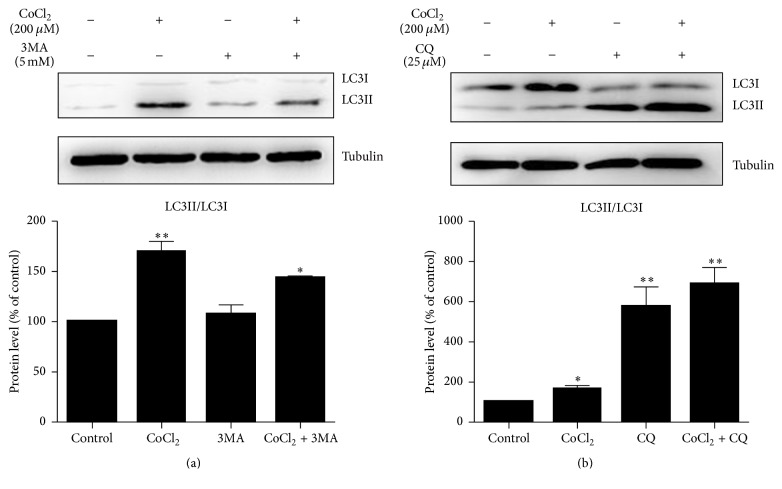
3MA and CQ inhibited autophagy induced by CoCl_2_. Western blotting analysis revealed the protein level of LC3II/LC3Ι treated with 3MA (a) or CQ (b) in the presence and absence of CoCl_2_ in C2C12 cells. The bands were quantified using Image J and the expression levels of LC3II/LC3Ι were normalized relative to tubulin. ^*∗*^*P* < 0.05 and ^*∗∗*^*P* < 0.01 compared to control group.

To further verify the real effects of CoCl_2_-induced autophagy in C2C12 cells, we utilized 3MA (5 mM) and CQ (25 *μ*M) to inhibit autophagy. On the one hand, 3MA inhibited autophagy-dependent protein degradation and we found that 3MA significantly decreased the ratio of LC3II/LC3I (Figure 2(a)). On the other hand, CQ treatment for 6 h increased in LC3B-II protein ensured that the observed increase in LC3B-II protein was due to increased autophagic flux (Figure 2(b)).

### 3.3. Autophagy Signal Pathways Were Activated by Cobalt Chloride

**Figure 3:**
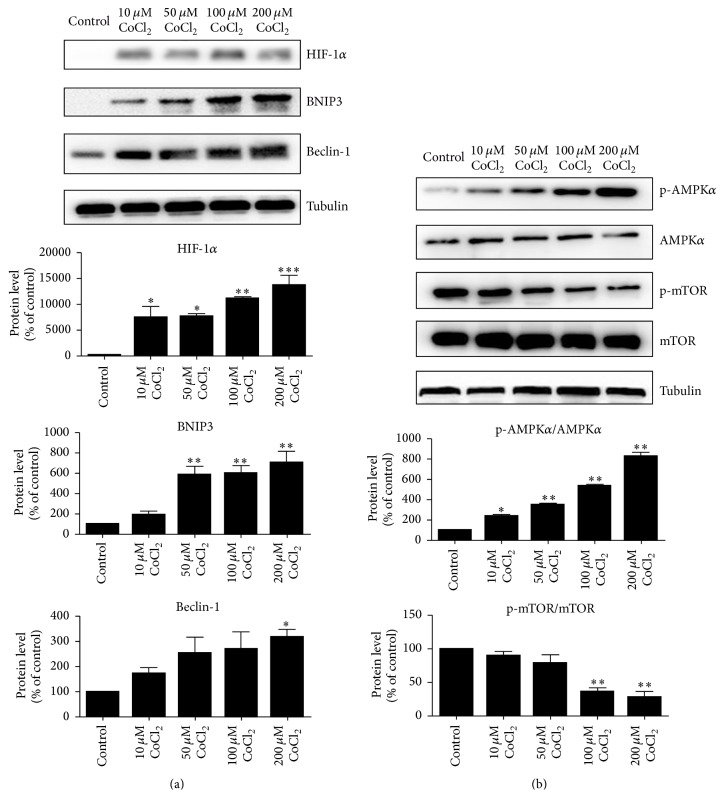
CoCl_2_ induced autophagy via the HIF-1*α*/BNIP3/beclin-1 and AMPK*α*/mTOR pathways. Western blotting analysis revealed the protein levels of HIF-1*α*/BNIP3/beclin-1 (a) and p-AMPK*α*/AMPK*α* and p-mTOR/mTOR (b) in C2C12 cells treated with CoCl_2_. The bands were quantified using Image J and the expression levels of proteins were normalized relative to tubulin. ^*∗*^*P* < 0.05, ^*∗∗*^*P* < 0.01, and ^*∗∗∗*^*P* < 0.001 compared to control group.

To understand the mechanisms underlying the autophagy in C2C12 cells under hypoxic conditions, we next evaluated the protein expression of HIF-1*α* and its downstream target, BNIP3. The results indicated upregulation of HIF-1*α* and BNIP3 with CoCl_2_ treatment, suggesting the involvement of the HIF-1*α*/BNIP3 signaling pathway in CoCl_2_-induced autophagy. Furthermore, compared with control group, beclin-1 increased in a concentration-dependent manner (Figure 3(a)). AMPK activation or mTOR inhibition resulted in autophagy. As expected, p-AMPK*α* level elevated upon induction of CoCl_2_ and the ratio of p-AMPK*α*/AMPK*α* was increased significantly. Oppositely, the ratio of p-mTOR/mTOR was gradually decreased with concentrations (Figure 3(b)).

### 3.4. Cobalt Chloride Induced C2C12 Cells Protein Degradation

**Figure 4:**
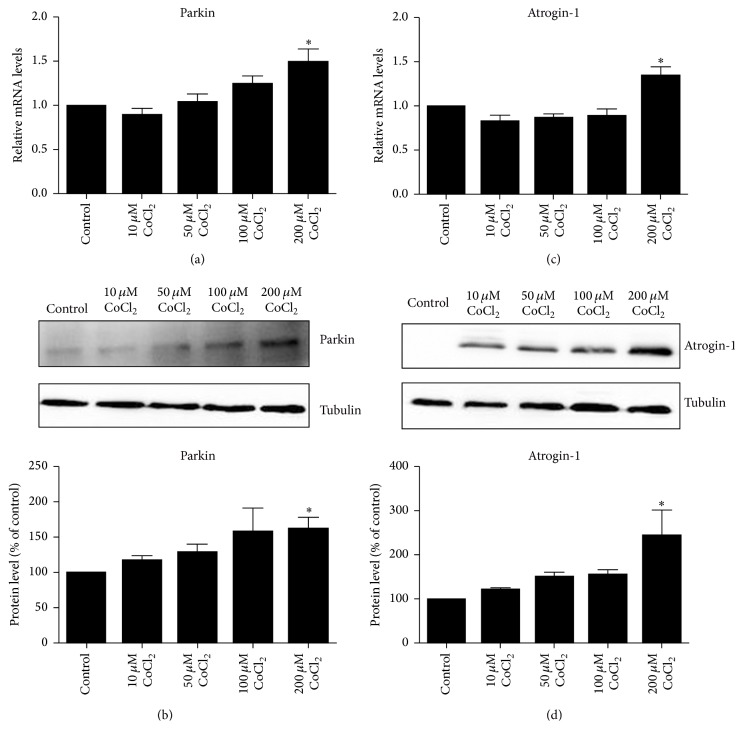
CoCl_2_ promoted C2C12 cells protein degradation. QRT-PCR and Western blotting were used to determine the mRNA and protein levels of parkin (a and b) and atrogin-1 (c and d) in C2C12 cells treated with CoCl_2_. The bands were quantified using Image J and the expression levels of parkin and atrogin-1 were normalized relative to tubulin. ^*∗*^*P* < 0.05 compared to control group.

Parkin is one of autophagy-related E3 ligases. Our results demonstrated that parkin was significantly increased in CoCl_2_ groups compared to control group (Figures 4(a) and 4(b)). The level of atrogin-1, a muscle-specific ubiquitin ligase that mediates the degradation of muscle protein, was elevated by CoCl_2_ in a concentration-dependent manner (Figures 4(c) and 4(d)).

### 3.5. Inhibition of Autophagy Induced by Cobalt Chloride Promoted Cell Survival in C2C12 Myotubes

**Figure 5:**
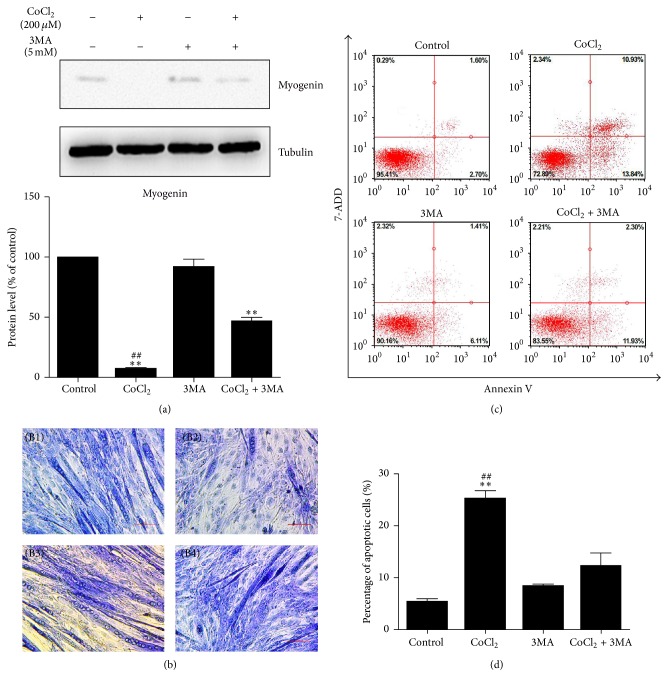
Inhibition of autophagy induced by cobalt chloride promoted cell survival in C2C12 myotubes. (a) Western blotting result showed the protein level of C2C12 myotubes treated with CoCl_2_ with/without 3MA. The bands were quantified by Image J and the expression level of myogenin was normalized relative to tubulin. (b) Giemsa staining images showing morphology changes in C2C12 cells. Pictures were taken at the same magnification (40x). Scale bar: 100 *μ*m. (B1) Control group, (B2) 200 *μ*M CoCl_2_ group, (B3) 5 mM 3MA group, and (B4) 200 *μ*M CoCl_2_ + 5 mM 3MA group. (c, d) Flow cytometry for apoptosis stained with Annexin V-FITC on the *x*-axis and 7-ADD on the *y*-axis. ^*∗∗*^*P* < 0.01 compared to control group; ^##^*P* < 0.01 compared to CoCl_2_ + 3MA group.

Western blotting analysis revealed the expression of myogenin was recovered with 3MA treatment (Figure 5(a)). In favor of increasing of myogenin protein in 3MA + CoCl_2_ group, Giemsa staining images showed more spindly ring-shaped myotubes formation in 3MA + CoCl_2_ group compared with CoCl_2_ group (Figure 5(b)). The results of flow cytometry showed that the percentage of cells undergoing apoptosis in response to CoCl_2_ treatment was 25.21%, while 3MA had a positive effect on C2C12 survival under hypoxia conditions, and the percentage of apoptosis was significantly decreased by 13.11% when cocultured with 3MA (Figure 5(c)). Overall, these data suggested that inhibition of autophagy played a role in counteracting atrophy in vitro and had a positive effect on C2C12 cells development.

## 4. Discussion

Hypoxia-induced cell damage has been studied in various cell types. In this study, we exposed C2C12 myotubes to different concentrations of CoCl_2_, a well-known hypoxia-mimetic agent that competes with the activity of bivalent ions and suppresses the formation of oxygenated hemoglobin [12]. In cell culture systems, CoCl_2_ blocks the catalysis of prolyl hydroxylases, leading to an intracellular hypoxia-like state [13, 14]. In our study, we found that the ratio of LC3-II/LC3-I was upregulated while the level of p62 was downregulated in C2C12 myotubes by CoCl_2_-induced hypoxia. An increase in LC3-II protein is considered a marker for elevated autophagosome formation, and a decrease in p62 can be interpreted as an increase in autophagy flux [15]. 3MA and CQ in the presence and absence of CoCl_2_ were used to verify the real effects of CoCl_2_ in autophagy.

In the present study, CoCl_2_ treatment resulted in the accumulation of HIF-1*α* protein. HIF-1*α* protein is located in the cytoplasm under normoxic conditions and can be ubiquitinated by Von Hippel Lindau (VHL) E3 ubiquitin ligase, thereby promoting protein degradation. Under conditions of hypoxia, proline residues of the oxygen-dependent degradation domain of HIF-1*α* are not hydroxylated due to the lack of sufficient amounts of O_2_. Therefore, pVHL cannot interact with HIF-1*α*, and finally the monomer remains in the cytoplasm and migrates to the nucleus, binding to constantly expressed *β*-monomer and forming the HIF-1*α* transcription factor [16]. BNIP3 contains a hypoxia response element (HRE) and appears to be a direct target of transcriptional activation by HIF-1 [10]; this molecule was originally reported to function as a BH3-only protein that induced programmed cell death [17]. More recently, BNIP3 has been reported to regulate autophagy through its interaction with LC3-related molecules at nascent phagophores [18, 19]. Bellot reported that the expression of BNIP3 is required for the optimal induction of autophagy under conditions of hypoxia [20]. Beclin-1 is a key protein involved in nucleus complex formation and creates a section of double membrane [8], which could be released by BNIP3 through hindering interaction with Bcl-2 and beclin-1. The result of the present study indicated that the expression of beclin-1 was increased by CoCl_2_ in a concentration-dependent manner and that induced the following autophagic process.

The process of autophagy involves complex autophagy regulating pathways. AMPK/mTOR is one of the most studied signal pathways of autophagy. AMPK activation leads to the inhibition of mTORC1 and its subsequent dissociation from ULK1 complex. A recent study reported that ULK1 was found to combine and to be phosphorylated by mTOR in palmitate induced insulin-resistant C2C12 myotubes. AMPK activation triggered a progressive reduction of mTOR activity and showed a protective effect against palmitate induced insulin resistance [21]. In addition, it was reported that AMPK activation is required to guarantee a proper autophagy-induced catabolism during long-term resistance exercise [22]. However, little is known about whether AMPK/mTOR signal involves CoCl_2_-induced autophagy. Our current results provided evidence that AMPK activation stimulated autophagy in C2C12 myotubes with CoCl_2_, through the suppression of mTOR phosphorylation.

Previous reports have shown that autophagy is a dynamic catabolic process that is involved in a wide range of physiological processes and the pathogenesis of diverse diseases. Parkin is the central ubiquitin ligase to autophagy pathways [23]. Interestingly, an autophagy deficiency in denervated slow-twitch soleus muscles delayed skeletal muscle atrophy and reduced mitochondrial activity and parkin expression [24]. Atrogin-1 was identified to be specific to muscle atrophy and to be involved in targeting important muscular signaling pathways for protein degradation [25]. Our results showed that parkin and atrogin-1 were upregulated by CoCl_2_ in a dose-dependent manner, which indicated that CoCl_2_-induced hypoxia could facilitate myofibrillar degradation.

To determine the effect of CoCl_2_-mimicked hypoxia-induced autophagy in myogenesis, we suppressed the activation of autophagy by 3MA, leading to the upregulation of myogenin as well as impaired myoblast fusion compared with that in the CoCl_2_ group, suggesting that hypoxia-induced autophagy has a negative role in muscle differentiation. Maintenance of muscle mass depends not only on myogenesis but also on the number of muscle fibers. Numerous studies have demonstrated that autophagy has a protective effect against apoptosis under conditions of oxidative stress. In contrast, some studies suggested that autophagy facilitated apoptosis with necrotic morphology and autophagosome formation [26–28]. To clarify further the functional significance of autophagy in C2C12 cells, we used flow cytometry to detect cell apoptosis, and the results indicated that the percentage of apoptotic cells was increased by culture in the presence of CoCl_2_, while inhibition of autophagy by 3MA had a protective effect against apoptosis under hypoxic conditions. These results indicated that hypoxia-induced autophagy facilitated apoptosis and myofibrillar degradation.

In conclusion, the findings of the present study demonstrated that CoCl_2_-mimicked hypoxia induced autophagy via the HIF-1*α*/BNIP3/beclin-1 and AMPK/mTOR signaling pathways. Excessive hypoxia-induced autophagy has a myotoxic effect on C2C12 myotubes and may provide a potential therapeutic target in muscle wasting disorders.
